# Dabigatran antagonizes growth, cell‐cycle progression, migration, and endothelial tube formation induced by thrombin in breast and glioblastoma cell lines

**DOI:** 10.1002/cam4.857

**Published:** 2016-09-07

**Authors:** Fabrizio Vianello, Luisa Sambado, Ashley Goss, Fabrizio Fabris, Paolo Prandoni

**Affiliations:** ^1^ Department of MedicineUniversity of Padova School of MedicinePadovaItaly; ^2^ Boehringer Ingelheim International GmbHIngelheim am RheinDeutschlandGermany; ^3^ Department of CardiologicThoracic and Vascular SciencesUniversity of Padova School of MedicinePadovaItaly

**Keywords:** Cancer, dabigatran, PAR‐1, thrombin

## Abstract

Thrombin activates its G‐coupled seven transmembrane protease‐activated receptor (PAR‐1) by cleaving the receptor's N‐terminal end. In several human cancers, PAR1 expression and activation correlates with tumor progression and metastatization. This provides compelling evidence for the effectiveness of an appropriate antithrombin agent for the adjuvant treatment of patients with cancer. Dabigatran is a selective direct thrombin inhibitor that reversibly binds to thrombin. In this study, we aimed to explore if dabigatran may affect mechanisms favoring tumor growth by interfering with thrombin‐induced PAR‐1 activation.

We confirmed that exposure of tumor cells to thrombin significantly increased cell proliferation and this was coupled with downregulation of p27 and concomitant induction of cyclin D1. Dabigatran was consistently effective in antagonizing thrombin‐induced proliferation as well as it restored the baseline pattern of cell cycle protein expression. Thrombin significantly upregulated the expression of proangiogenetic proteins like Twist and GRO‐*α* in human umbilical vascular endothelial cells (HUVEC) cells and their expression was significantly brought down to control levels when dabigatran was added to culture. We also found that the chemoattractant effect of thrombin on tumor cells was lost in the presence of dabigatran, and that the thrombin antagonist was effective in dampening vascular tube formation induced by thrombin. Our data support a role of thrombin in inducing the proliferation, migration, and proangiogenetic effects of tumor cells in vitro. Dabigatran has activity in antagonizing all these effects, thereby impairing tumor growth and progression. In vivo models may help to understand the relevance of this pathway.

## Introduction

Advanced malignancy often correlates with activation of the coagulation system, termed cancer coagulopathy, which is associated with increased mortality rates 1. Several coagulation factors play a pathogenetic role in the induction of such a hypercoagulable state of cancer 2. Besides increasing the risk of thrombosis of cancer patients, the hypercoagulable state fuels critical cellular events in the tumor microenvironment, including cell proliferation, cell adhesion, angiogenesis, and invasion. Based on this two‐way relationship between cancer and venous thromboembolism, preclinical studies have addressed the question whether anticoagulants like heparin may have antineoplastic properties, particularly in aggressive cancers like glioblastoma and breast carcinomas 3, 4. Although a direct antiproliferative effect of heparins has not been consistently observed, other mechanisms have been identified which interferes with processes such as angiogenesis, migration, and metastatization of cancer cells. Extracellular vesicles (EV), structures with stimulating effect on neovascularization and tumor cell growth, are not allowed to attach to glioblastoma cells by heparin, which could possibly result in an antitumor effect 5. Mechanisms like prevention of tube‐like structure formation in glioblastomas are also affected by heparin, which suggests the ability of heparin to inhibit early steps of angiogenesis 6. Antiangiogenetic effects of heparin have been shown also in breast cancers. In fact, heparin impairs the growth of MDA‐MB‐231 breast cancer cells by interfering with thrombin to reverse the stimulatory effect of thrombin on angiogenesis 7. Short‐chain length oligosaccharide derived from polymeric heparin have been found capable of inhibiting the migration of MDA‐MB 231 breast cancer cells in vitro and limiting the growth of secondary tumors in vivo 4. More recently, not only heparins but also the Xa inhibitor fondaparinux has showed to dramatically reduce the proangiogenic potential of platelets, thereby limiting platelet‐driven metastatization of breast cancer cells 8. Among other anticoagulants with antineoplastic properties, warfarin has shown to reduce the metastatization of mammary rat carcinomas to the lung 9. No preclinical data on cancer progression are available for glioblastoma and warfarin.

In spite of somehow convincing preclinical data on the antitumor properties of heparin and other anticoagulants, evidences of a significative impact on the survival of cancer patients are still lacking 10, 11. The fact that cancer‐related effects of heparins do not have a precise mechanism of action hampers the possibility to define the subset of cancer whose growth has more chances to be affected by heparins.

Thrombin is the main serine protease regulating the coagulation cascade. Through activation of protease‐activated receptor‐1 (PAR‐1), thrombin exerts tumor‐enhancing effects in many human malignancies 12. Targeting thrombin with the aim of developing novel anticoagulants has led to the discovery of dabigatran, a selective direct thrombin inhibitor currently prescribed to patients with atrial fibrillation or venous thromboembolism 13. With this background on the role of thrombin in tumor cell progression, there is need to address the question whether, by displacing thrombin from PAR‐1‐binding, dabigatran may be favorably exploited to uncouple thrombin‐driven mechanisms promoting tumor growth. In this paper, we first validate the previously reported cancer‐promoting effects of thrombin and then we sought to evaluate the antagonism properties of dabigatran.

## Methods

### Cell cultures and drug treatments

The human breast carcinoma cell line MDA‐MB231 and the human glioblastoma U87‐MG were kindly provided by Prof. Rosato and Prof. Basso of this institution. Both cell lines have been showed to express PAR‐1 at high level.

Cell lines were maintained in DMEM supplemented with 10% FBS and 2 mmol/L l‐glutamine and 1% of penicillin‐streptomycin (all from Euroclone, Milan, Italy). Culture media of MDA‐MB231 cells was supplemented with 1% HEPES (Sigma‐Aldrich S.r.l., Milan, Italy). Human umbilical vascular endothelial cells (HUVEC) were purchased from Promocell GmbH (Heidelberg, Germany) and were maintained in Endothelial cell growth Basal Medium‐2 supplemented with 10% FBS and 1% penicillin‐streptomycin. All cell lines were grown in 37°C in CO_2_. Tumoral and HUVEC cells were starved for 72 or 12 h, respectively. Thrombin (Sigma‐Aldrich S.r.l) was used at different concentration from 0.1 U/mL to 1 U/mL. Dabigatran (kindly provided by Boehringer Ingelheim, Ingelheim am Rhein, Germany) was used at the concentrations of 100, 500, or 1000 nmol/L following reconstitution in DMSO and HCl 1 mol/L.

### Proliferation assay

Proliferation induced by thrombin was quantitated by Cell titer 96 Aqueous One solution Cell Proliferation assay kit (Promega, Milan, Italy) according to supplier's protocol. The method is based on the reduction of MTS tetrazolium compound by viable cells to generate a colored formazan product that is soluble in cell culture media. The formazan dye produced by viable cells can be quantified by measuring the absorbance. Briefly, after starvation, cells were seeded at the concentration of 1 × 10^4^ in 96‐well plates at different conditions and incubation time. MTS was then added to each well and plates incubated for 2 h at 37°C. Plates were read at 490 nm of absorbance using a Wallac Victor 2 Counter Plate Reader (PerkinElmer Life and Analytical Sciences, Milan, Italy). Each experiment was made in triplicate. Raw data were expressed as fold increase proliferation compared to untreated samples.

### Western blotting

Following starving and treatment, cells were harvested, lysed with 2X Laemmli sample buffer, and boiled for 10 min. SDS electrophoresis was performed on 7.5% polyacrylamide gels run at 120 mA (RT for 45 min). Proteins were then transferred to nitrocellulose membranes (Biorad Laboratories, Hercules, CA) at 350 mA at RT for 2 h. Residual binding sites were blocked for 1 h at RT in TBST/3% low‐fat milk. Membranes were washed three times with TBST and incubated over night at 4°C with the following primary antibodies (1 *μ*g/mL): p27, Cyclin D1, Twist, Tubulin (all from SantaCruz Biotechnology Inc. Heidelberg, Germany), Gro‐*α* (Abcam, Cambridge, UK).

After incubation with anti‐mouse or anti‐rabbit secondary HRP‐conjugated antibodies (1:2000 and 1:10000, respectively, from Sigma), membranes were washed and treated with enhanced LumiGLO chemiluminescence reagents (KPL, Gaithersburg, Maryland, USA) before exposure to X‐ray film.

Following acquisition using a CCD camera in a light table with shading correction, densitometric analysis was performed by ImageJ 1.38 (Windows version of NIH Image, (http://rsbweb.nih.gov/ij/) and background correction was done with the default settings (rolling ball radius = 50).

### Rt‐pcr

RNA expression was analyzed using one‐step SYBR Green 1‐based real‐time RT‐PCR. Briefly, cells (5 × 10^6^) were harvested and centrifuged. Triazol (Life technology, Thermo Fisher Scientific) Waltham, Massachusetts, USA) 1 mL was added to pellet and cells were lysed by repetitive pipetting. After 5 min incubation, 0.2 mL chloroform was added and samples were incubated for 3 min at RT. After centrifugation, the upper phase was transferred and an equal volume di 70% ethanol was added. RNA suspension was then washed and RNA was resuspended in RNA‐free water (PureLink^®^ RNA Mini Kit, Life Technology). RNA was quantitated by Qubit^™^ RNA HS Assay (Thermo Fisher Scientific Inc., Waltham, MA).

cDNA was synthetized from 1 *μ*g RNA by the SuperScript^®^ VILO^™^ cDNA Synthesis Kit (Thermo Fisher Scientific Inc.). Template‐diluted cDNA was amplified using the ABI Prism 7900HT Sequence Detection System (Thermo Fisher Scientific Inc) in 20 *μ*L. Results were analyzed with Sds 2.4 software (Thermo Fisher Scientific Inc). All the experiments were run in duplicate. The primer sets were as follow:

*β*‐Actin: forward 5′ GGGACGACATGGAGAAAATCTG 3′, reverse 5′ CACGCAGCTCATTGTAGAAGGT 3′;

Gro‐*α*: forward 5′ TTCACCCCAAGAACATCCAAAG 3′, reverse 5′ CAAACACATTAGGCACAATCCAGG 3′;

Twist: forward 5′ GGAGTCCGCAGTCTTACGAG 3′ reverse 5′ TCTGGAGGACCTGGTAGAGG 3′.

Twist and Gro‐*α* levels were normalized using the ΔΔC_t_ method.

### FACS analysis of cell cycle

For this purpose, U87‐MG cells were treated for 24 h at different conditions. A duration of 30 min before the end of incubation, 100 *μ*mol/L BrdU (Merck S.p.a, Milan, Italy) was added and cells were incubated for 1 h, then harvested, fixed in ice‐cold 70% ethanol overnight. Cells were then incubated in freshly prepared 2 mol/L HCl for 30 min, and then in 0.1 mol/L sodium borate (pH 8.5) for 2 min. Cells were then resuspended in dilution buffer (1×PBS, 0.5% Tween 20 and 0.5% BSA) with 0.3 *μ*g of anti‐BrdU antibody 1:50 (LifeSpan Bioscience, Seattle, WA) and incubated at 4°C for 1 h in the dark, followed by an incubation with 0.125 *μ*g of FITC‐conjugated goat anti‐mouse IgG (Sigma Aldrich, 1:100) in the dark at 4°C for 1 h. Finally, cells were incubated with 10 *μ*g/mL propidium iodide/1×PBS for 15 min in the dark. Samples were analyzed by flow cytometry using a FC500 cytofluorimeter (BD Biosciences, Milan, Italy) and data were analyzed by FlowJo.

### Endothelial cord formation

Matrigel (BD Bioscience) was thawed and liquefied on ice, and then 50 *μ*L of Matrigel was plated to 96‐well plates at a horizontal level that allows the Matrigel to distribute evenly, and incubated for 30 min at 37°C. HUVEC cells (5 × 10^4^) were resuspended with Endothelial cell growth Basal Medium (EBM‐2, Carlo Erba Reagents, Milan, Italy), and loaded on the top of the Matrigel. Different concentrations of experimental compounds were then added. Following incubation (24 h at 37°, 5% CO_2_), four random pictures per well were captured with Leica microscope and the number of branch and nodes were quantitated by image J software with black markers highlighting individual branches. All the experiments were made in duplicate.

### Chemotaxis assay

Briefly, starved cells growing at confluent phase were trypsinized and 5 × 10^4^ cells were suspended in 200 *μ*L of 0.5% FCS medium in the upper chamber of a Boyden transmigration system. The lower chamber was filled with 0.5% FCS medium supplemented with the experimental compounds.

After 4 h incubation (37°C–5% CO_2_), inserts were removed and cells that had not invaded were removed from the upper face of the filters using cotton swabs, and cells that had invaded to the lower surface of the filters were fixed for 30 min in 4% paraformaldehyde and stained with crystal violet for 5 min (Sigma). The filters were then mounted with Eukitt (Bio Optica, Milan, Italy) on glass slides. Results were expressed as mean number of cells in 5 random fields captured with a Leica microscope at 10× (total magnification 100×). Images were analyzed with ImageJ software. Briefly, after loading the images, we first adjusted the color threshold using the default thresholding method (Image→AdjustColor→ Threshold→select). We then analyzed the images using Analyze→Analyze particle→summarize.

### Statistical analysis

Data were presented as the mean **± **SD. Differences between two groups of data were analyzed by nonparametric test (Mann–Whitney). All of the above analyses were conducted using GraphPad Software (La Jolla, CA).

## Results

### Effect of thrombin and dabigatran on tumor cell proliferation

**Figure 1:**
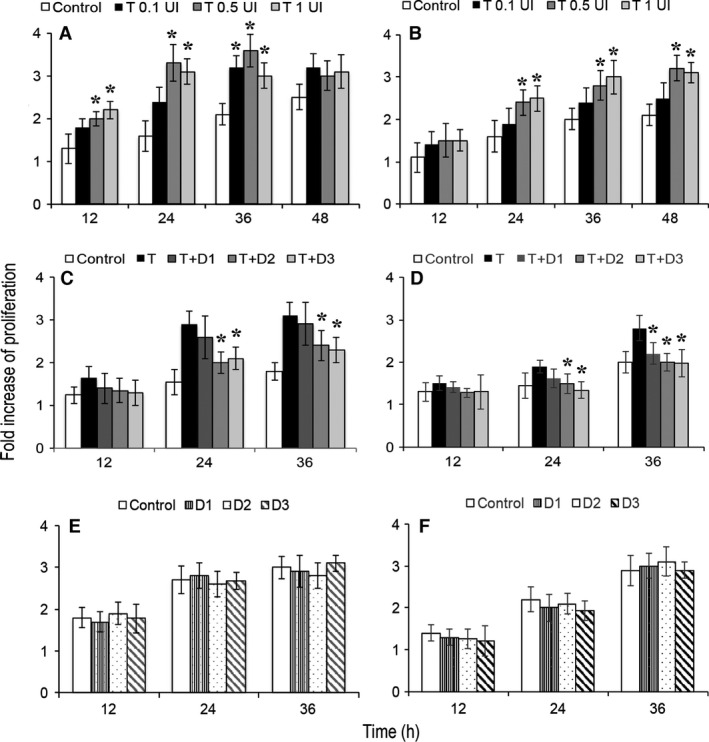
In vitro proliferation of MDA‐MB231 (A) and U87 (B) cells cultured with thrombin (A, B), with thrombin and dabigatran (C, D) and with dabigatran only. Proliferation was measured by measuring the absorbance of culture media following generation of formazan from reduction of MTS tetrazolium. The reaction is dependent on cell metabolism. Each condition had three replicates. Shown are the results of three experiments, with data expressed as mean fold increase of proliferation compared to T0. Error bars represent sd. T: thrombin 0.5 UI; D1: dabigatran 100 nmol/L; D2, dabigatran 500 nmol/L; D3, dabigatran 1000 nmol/L. *P < 0.05 (control vs. thrombin panels A,B or thrombin vs. thrombin/dabigatran panels C, D).

In agreement with previous data, flow cytometry with PAR‐1‐specific antibody confirmed high levels of PAR‐1 surface expression in glioblastoma and breast cancer cells (not shown) 14, 15. We then measured the proliferation of tumor cells in the presence of thrombin by means of a colorimetric assay assessing the direct correlation between metabolic activity and cell number. With this approach, we observed an increased proliferation of PAR‐1‐positive breast cancer cells (Fig. 1A) and glioblastoma cells (Fig. 1B) exposed to thrombin. A statistically significant difference (*P* < 0.05) was always observed with thrombin 0.5 or 1 UI, at 12‐24‐36 h in breast cancer cell cultures and at 24‐36‐48 h for glioblastoma cells. Thus, these data confirm previous findings that thrombin stimulates in vitro proliferation of PAR‐1‐expressing tumor cells.

We then tested whether dabigatran antagonizes tumor cell proliferation induced by thrombin. As shown in Figure 1 (C and D), dabigatran dampened tumor proliferation induced by thrombin in a concentration‐dependent manner that reached statistical significance at 500 and 1000 nmol/L both in breast cancer (Fig. 1C, *P* = 0.01 and 0.02 at 24 h and *P* = 0.046 and 0.025 at 36 h, respectively) and glioblastoma cells (Fig. 1D, *P* = 0.036 and *P* = 0.01 at 24 h; *P* = 0.014 and *P* = 0.026 at 36 h, respectively). No significative changes in proliferation were observed in cells treated with dabigatran only (Fig. 1E and F).

### Effect of thrombin and dabigatran on expression of proteins regulating cell cycle and angiogenesis

Having demonstrated a promoting effect of thrombin on cell proliferation, we next explored whether cell cycle progression may represent a mechanism modulated by the protease. In fact, there is evidence that thrombin is a growth factor that stimulates spontaneous mitogenesis by inducing activation of the cell cycle from G0 to G1 to S by downregulation of p27^Kip1^, following activation of PAR‐1 16. The cell cycle regulator p27Kip1 is an inhibitor of the G1 phase of the cell cycle, thereby acting as a tumor suppressor 17.

**Figure 2:**
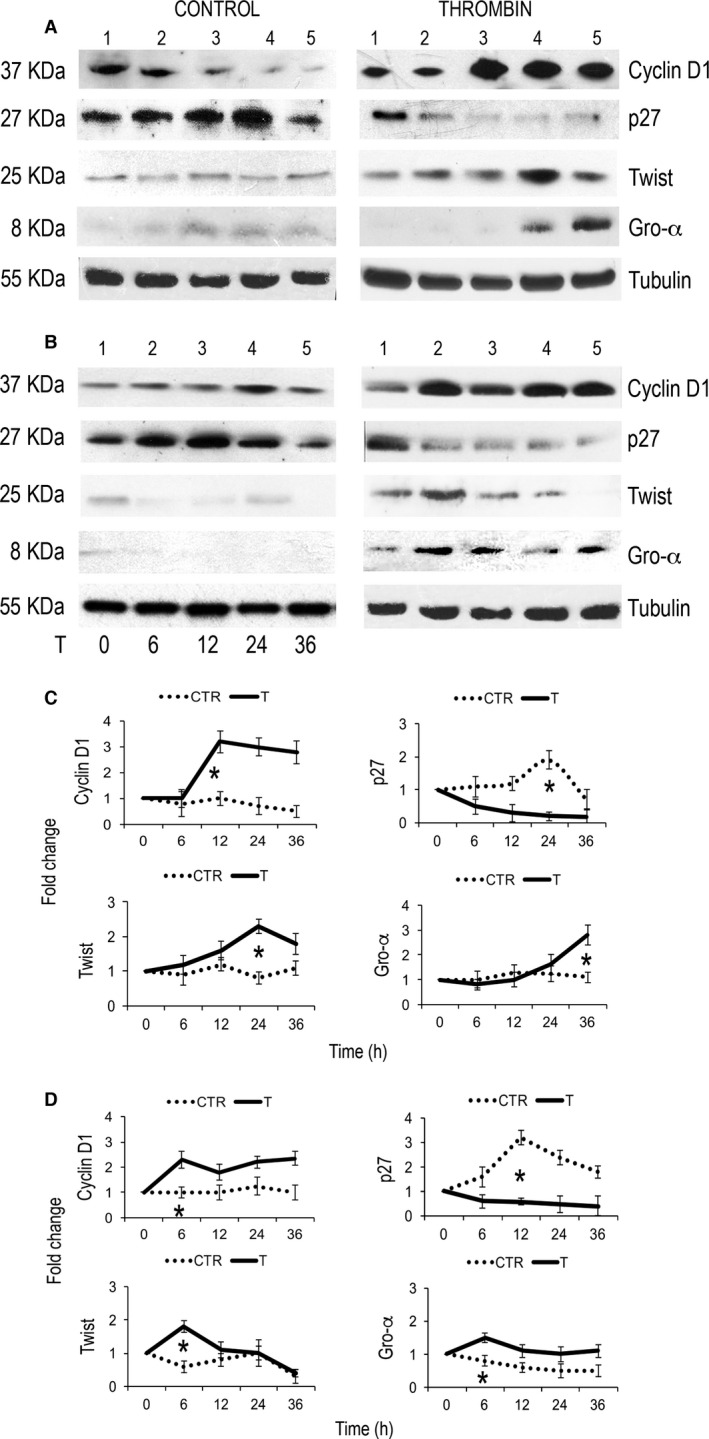
Western blot showing cyclin D1, p27, Twist, and Gro‐*α* level in MDA‐MB231 (A) and U87 cells (B) exposed to thrombin compared to untreated cells. (C, D) Cumulative results from two independent experiments with MDA‐MB231 (C) or U87 (D) cells are shown. *P < 0.05.

As shown in Figure 2, breast (A and C, left panel) and glioblastoma (B and D, left panel) cancer cells synchronized by serum starvation increased their expression of p27 by 6 to 24 h, whereas expression of cyclin D1 showed a progressive reduction in breast cancer cells (Fig. 2A, left panel) and a rather stable level of expression in glioblastoma cells (Fig. 2B, left panel). The addition of thrombin to cell cultures resulted in univocal and stable downregulation of p27 expression by 6 h (0.2 and 0.4‐fold decrease, *P* = 0.015 and *P* = 0.006 compared to unstimulated breast and glioblastoma cells, respectively; Fig. 2A and B, right panels) and the induction of cyclin D1 expression (up to 3.2‐fold increase; *P* = 0.028 and *P* = 0.044 compared to unstimulated breast and glioblastoma cells, respectively).

At the same extent, compared to untreated cells (Fig. 2A and B, left panel), thrombin was effective in inducing the expression of the angiogenetic proteins Twist and Gro‐*α* in breast cancer cells (Fig. 2A and C, right panel) and glioblastoma cells (Fig. 2B and D, right panel), with a similar pattern of upregulation for Twist (up to 2.3‐fold increase, *P* = 0.016 and *P* = 0.035 compared to unstimulated breast and glioblastoma cells, respectively) and a variable, yet significative Gro‐*α* kinetics (from 1.1 to 2.8 at 36 h for breast cancer cells and from 0.8‐ to 1.5‐fold increase at 6 h in glioblastoma cells, *P* = 0.037 and *P* = 0.045 compared to unstimulated breast and glioblastoma cells, respectively; Fig. 2). These results confirm that thrombin has a mitogenic as well as proangiogenetic effects on tumor cells expressing PAR‐1.

Having showed that thrombin affects proliferation and expression of proteins potentially involved in the enhancement of the malignant phenotype, we sought to test the efficacy of dabigatran, a selective thrombin inhibitor, in antagonizing these effects.

**Figure 3:**
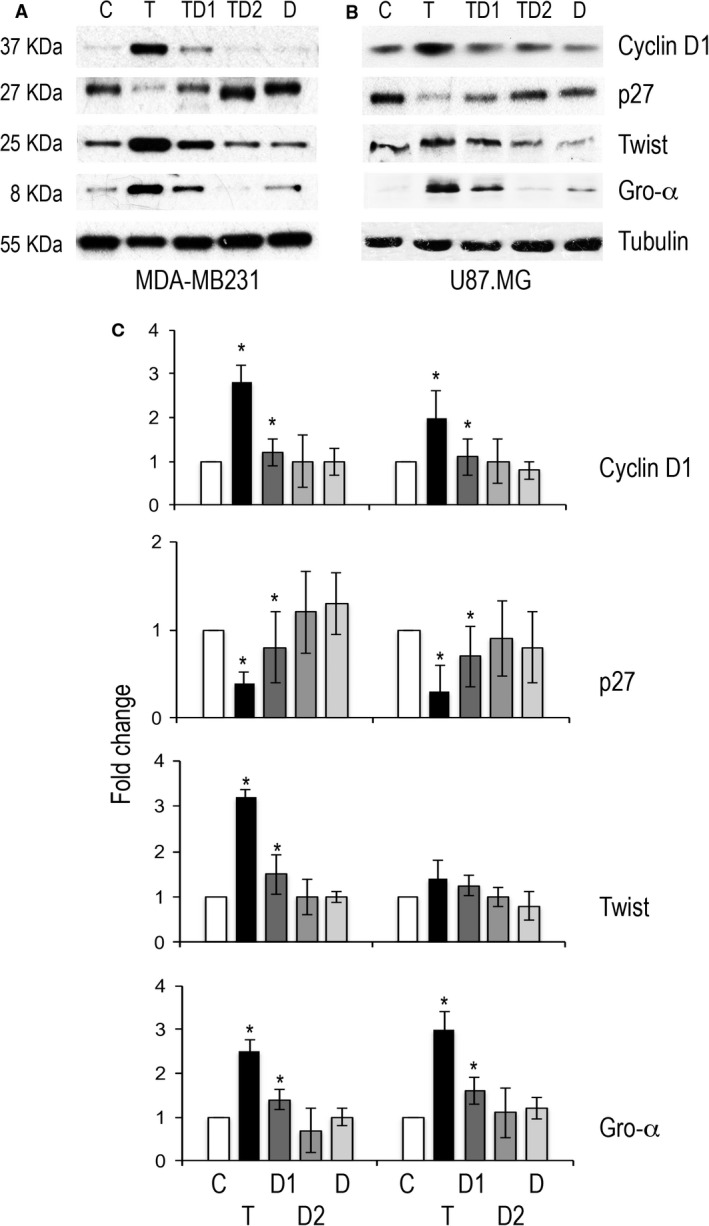
Western blot analysis of cyclin D1, p27, Twist, and Gro‐*α* level in MDA‐MB231 (A) and U87 (B) cells exposed to thrombin, thrombin and dabigatran or dabigatran. (C) Histograms showing cumulative results from two independent experiments. *P < 0.05.

As shown in Figure 3, downregulation of p27 and upregulation of cyclin D1 observed in breast cancer and glioblastoma cells cultured with thrombin was not observed in the presence of dabigatran The fold change difference of cells treated with the combination thrombin/dabigatran compared to cells exposed to thrombin was statistically significant in breast cancer cells (cyclin D1 expression fold change 2.8 vs. 1.2 for thrombin and thrombin/dabigatran, *P* = 0.022; p27 expression fold change 0.4 vs. 0.8 for thrombin vs. thrombin/dabigatran, *P* = 0.034; Fig. 3A and C) as well as with glioblastoma cells (cyclin D1 expression fold change 2 vs. 1.1 for thrombin and thrombin/dabigatran, *P* = 0.03; p27 expression fold change 0.3 vs. 0.7 for thrombin vs. thrombin/dabigatran, *P* = 0.028; Fig. 3B and C). The antagonizing effect of dabigatran was not always dependent on its concentration as in cyclin D 1 expression (Fig. 3A–B).

The ability of dabigatran to antagonize the effect of thrombin was significant also with regard to the expression of angiogenetic proteins. In fact, the upregulation of Twist and Gro‐*α* in breast cancer cells treated with thrombin (3.2 and 2.5‐fold increase, respectively) was not observed in the presence of dabigatran (1.5 and 1.4, *P* = 0.03 for both proteins; Fig. 3A and C). Glioblastoma cells showed a similar pattern of expression although the difference reached statistical significance only for Gro‐*α* (*P* = 0.04; Fig. 3B and C).

**Figure 4:**
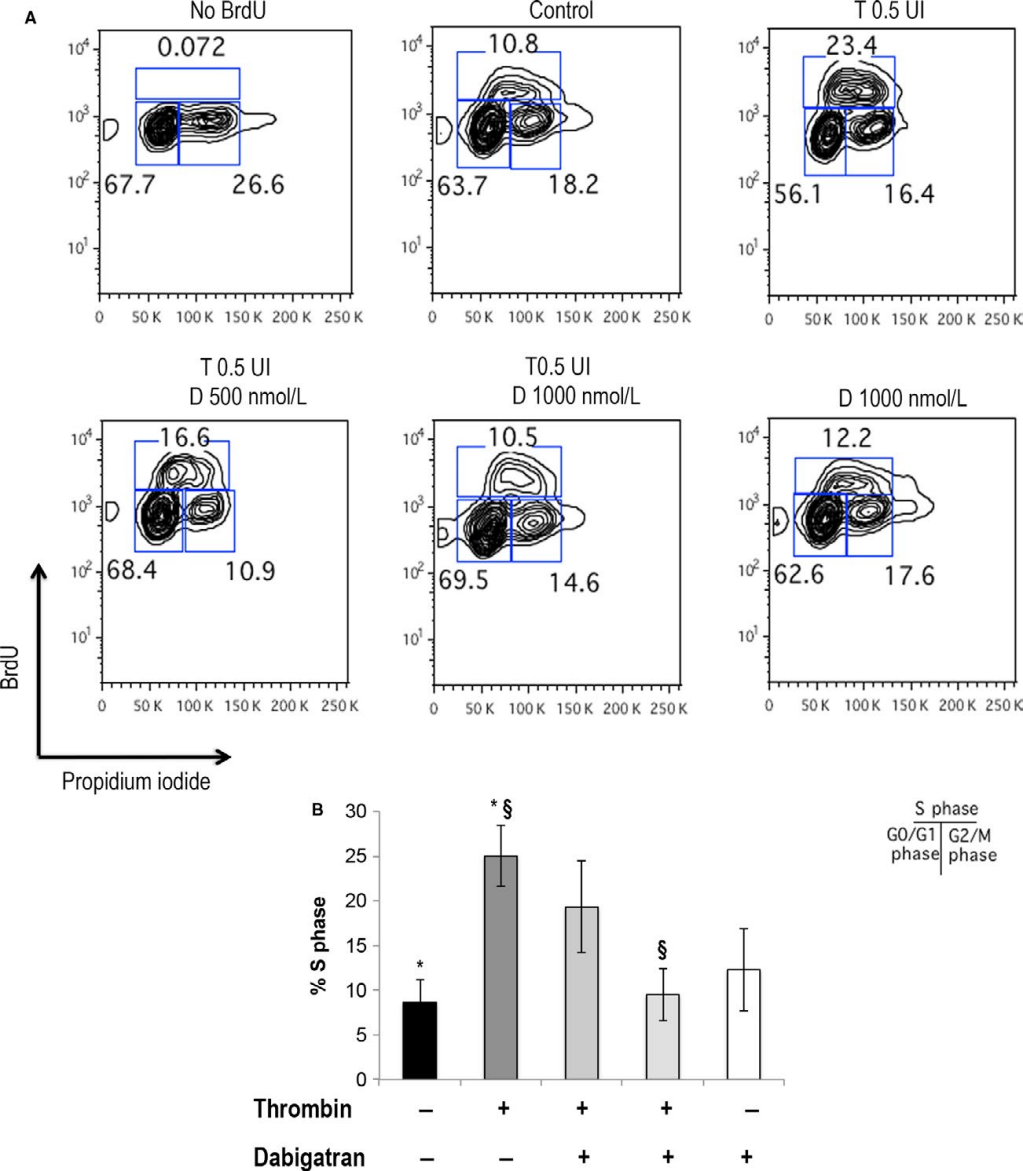
Cell cycle analysis of MDA‐MB231cells by BrdU. Panel A: MDA‐MB231cells were treated with thrombin, thrombin and dabigatran or dabigatran for 24 h and pulsed with BrdU for 1 h followed by staining with anti‐BrdU FITC and PI. BrdU‐labeled G0/G1, S, and G2/M populations are shown. Panel B: histograms of cells at S phase for each different treatment. *P < 0.05 control versus thrombin; ^§^ *P* < 0.05 thrombin versus thrombin/dabigatran.

Cell cycle progression in tumor cells exposed to thrombin and dabigatran was also evaluated by BrdU FACS analysis in breast cancer cells and results are shown in Figure 4. Exposure to thrombin was associated with an increased fraction of breast cancer cells in S phase compared to controls (24.7 ± 9.4% vs. 8.5 ± 2.6%, *P* = 0.033), but the effect of thrombin was no longer observed in the presence of dabigatran as S phase did not differ from controls (9.5 ± 2.6 vs. 8.6 ± 2.9, *P* = ns). Statistical significance between thrombin and thrombin/dabigatran was reached only with the thrombin inhibitor at 1000 nmol/L (*P* = 0.038, Fig. 4B).

**Figure 5:**
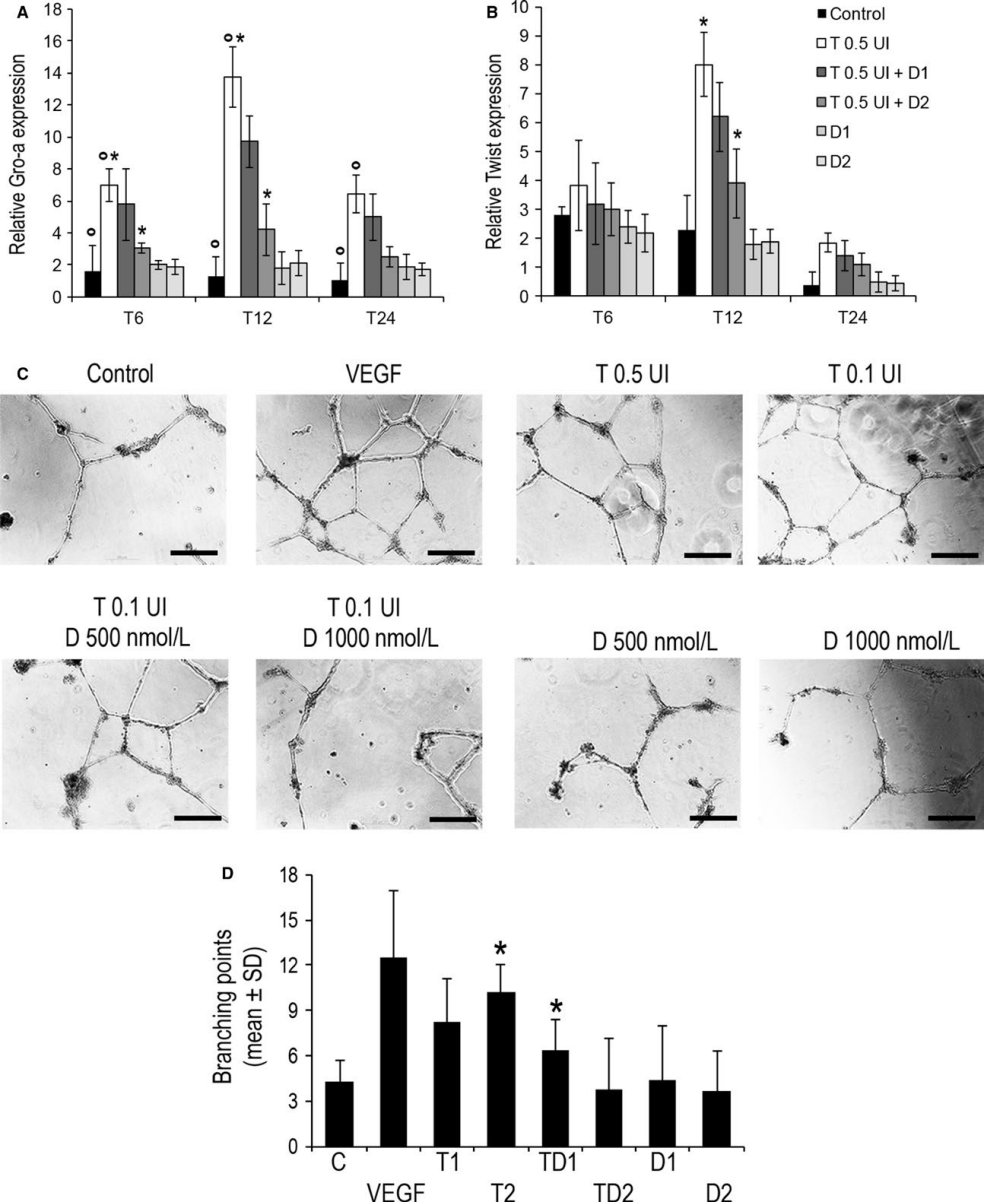
Angiogenetic properties of thrombin and dabigatran in Human umbilical vascular endothelial cells (HUVEC) cells. (A) mRNA expression of Gro‐*α* and Twist measured by real‐time PCR. Relative expression normalized to *β*‐actin is shown. (B) Effect of thrombin and dabigatran on tube formation by (HUVECs). Cells were cultured on Matrigel with medium containing 2% serum. Representative photomicrographs (×100, C) and quantitation (D) of branching points are shown. *P < 0.05. Scale bar, 250 *μ*m.

Thrombin‐induced angiogenesis entails endothelial cell migration and tube formation 18. We then studied the role of thrombin and dabigatran in this fundamental process sustaining tumor progression. As shown in Figure 5, culture of HUVEC cells with thrombin induced upregulation of mRNA for Gro‐*α* (13.7 ± 2.9 fold‐increase at 12 h, *P* = 0.031 vs. control; Fig. 5A) and Twist (8 ± 1.1 fold‐increase at 12 h, *P* = 0.014 vs. control; Fig. 5B). On the contrary, thrombin‐induced mRNA expression was progressively brought down to normal levels when cells were exposed to dabigatran at different concentrations (*P* = 0.045 and *P* = 0.033 thrombin vs. thrombin dabigatran at 12 h Twist and Gro‐*α*, respectively; Fig. 5 A and B).

The expression of angiogenetic proteins in HUVEC cells was coupled to a twofold increase vascular tube formation in cells treated with thrombin (10.2 ± 4.4 branching points) as measured compared to control (4.25 ± 1.35; *P* < 0.001; Fig. 5D). Interestingly, the ability of thrombin to increase tube formation was inversely correlated with its concentration. As observed for protein expression, the induction of tube formation was progressively lost when cells were incubated with thrombin and dabigatran (6.35 ± 2.5 branching points; *P* < 0.001 (Fig. 5C and D).

**Figure 6:**
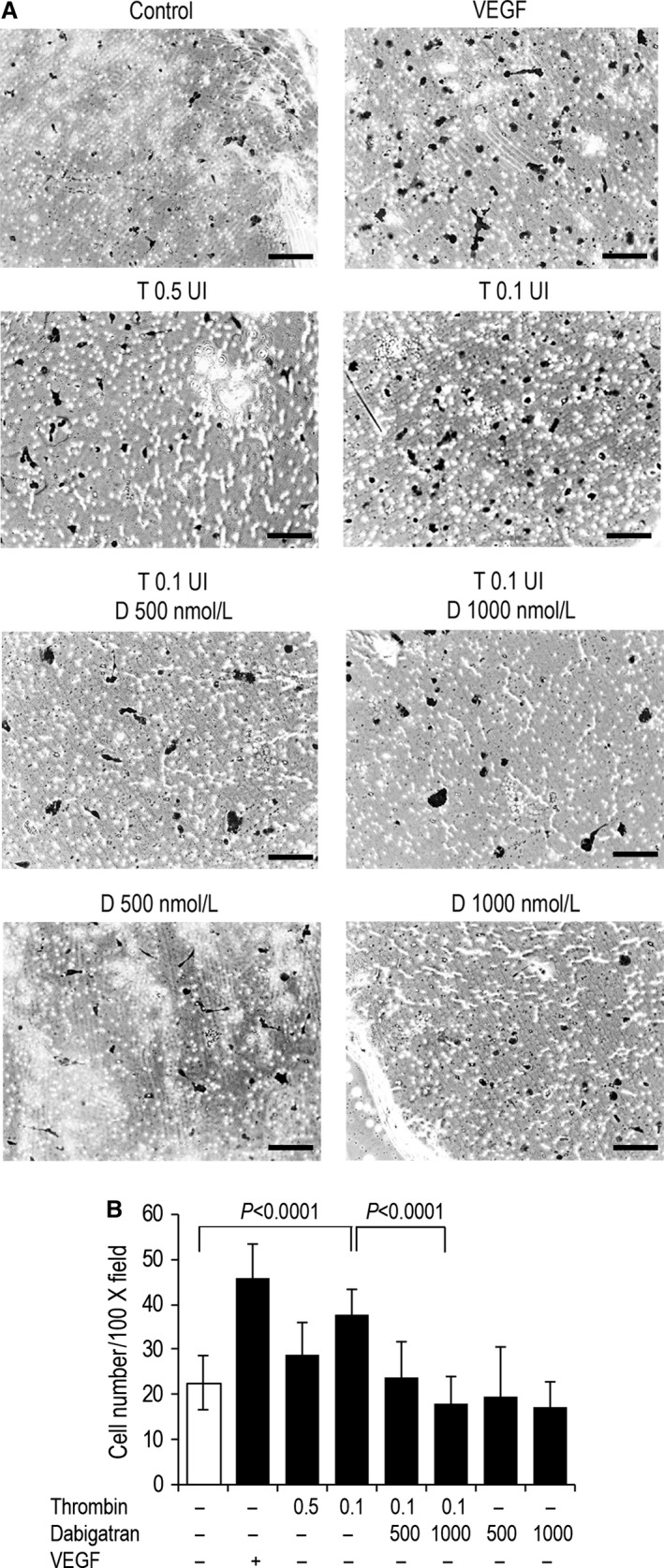
Dabigatran antagonizes thrombin‐driven chemotaxis of MDA‐MB231. Cells were seeded in the upper chamber of a Boyden transmigration system. After 4 h incubation, cells migrated to the lower surface of the filters were fixed, stained, and quantitated. Representative pictures (A) and the average number of cells counted in five microscopy fields (B, ×100) are shown. Scale bar, 100 *μ*m.

Cell invasion is facilitated by the acquisition of motility and PAR___1 activation has been shown to play a pivotal role on this matter 19. We then tested the efficacy of thrombin as chemotactic agent for tumor cells and the role of dabigatran as a potential antagonist of cell motility in a Boyden chamber assay. As shown in Figure 6, thrombin exerted chemoattraction on breast cancer cells compare to control medium and motility was inversely correlated with thrombin concentration (29 ± 6.9 vs. 37.8 ± 5.7, respectively for thrombin 0.5 and 0.1 U/mL; *P* < 0.0001 thrombin 0.1 U/mL vs. control) (Fig. 6B). Co‐incubating tumor cells with thrombin and dabigatran significantly reduced migration through the filter compared to cells exposed to thrombin alone, and the reduction was dependent on dabigatran concentration (23.8 ± 8.1 and 17.5 ± 6.1 thrombin/dabigatran 500 and 1000 nmol/L, respectively; *P* < 0.0001 thrombin 0.1 U/mL vs. thrombin/dabigatran 1000) (Fig. 6A and B).

## Discussion

The central role of thrombin in the complex interplay between coagulation and cancer progression has been convincingly demonstrated 20, 21. The data presented in this study corroborate the ability of thrombin to increase proliferation, migration as well as to modulate neoangiogenesis of glioblastoma and breast cancer cells in an in vitro system. A major and novel observation from our study is the consistent ability of dabigatran to abolish all these factors promoting cancer growth and progression.

The stimulation of cell proliferation by thrombin in both normal and tumor cells is well established 12. In agreement with previous findings from different experimental models, we found that proliferation of breast cancer and glioblastoma cells is coupled with the induction of cyclin D1, an activator of kinases driving progression through the G1 phase of the cell cycle, and with downregulation of p27, a negative regulator of the cell cycle. Naldini et al. showed that thrombin causes a reversal of cyclin D1 downregulation and growth inhibition induced by IFN*γ* in U937 leukemia cells 22. Hu et al. provided data on p27 downregulation in prostate cancer cells treated with thrombin and they showed that downregulation was driven by micro‐RNA‐222 posttranscriptional regulation 16. Our data demonstrate that dabigatran reverses the associated molecular pattern of cyclin D1 induction and p27 downregulation triggered by thrombin. This is consistent with previous demonstration that cancer‐promoting effects of thrombin can be weakened by its potent and specific inhibitor, hirudin 16.

We found that thrombin is able to direct neoangiogenesis and that this is coupled with the upregulation of Twist, a multifaceted gene‐stimulating tumor migration and invasion 23. Our data are in agreement with Hu et al. who showed that thrombin upregulates Twist mRNA and protein 24. Twist upregulation is then responsible for induction of angiogenesis/growth factors like the chemokine GRO‐a, which we found upregulated as well.

Finally, we found that motility and migration of tumor cells is increased toward a gradient of thrombin. It has been previously demonstrated that thrombin, through PAR‐1 activation, influences the process of gastric cancer cell morphological change which in turn facilitates cell migration. The molecular basis of thrombin‐induced migration is in part explained by the ability of PAR‐1 to increase expression of myosin IIA and filamin B, both constituents of actin microfilament‐based cytoskeleton. Based on our and previous findings, the activation of PAR‐1 by thrombin may allow tumor cell to increase their motility and therefore to acquire a more metastatic phenotype. This increased motility of tumor cells may facilitate the extravasation toward a tissue gradient generated by other chemotactic molecules. Fibrin generation and the activation of thrombin receptors on platelets and endothelial cells are other proposed mechanisms by which thrombin may enhance metastasis 25. The antagonist effect of dabigatran may have relevance particularly in the context of intra‐and extravasation of tumor cells in the process of metastatization.

Other anticoagulants have shown antineoplastic activity in glioblastoma and breast cancer. Consistent data are available for unfractionated heparin (UH) and low‐molecular weight heparin (LMWH). Although the inhibitory activity of heparins in glioblastomas and breast cancer growth is multifactorial, some studies support a role of thrombin and PAR‐1. Balzarotti et al. found that growth of primary cell cultures of high‐grade gliomas was modestly yet significantly inhibited by enoxaparin and that this effect was dependent on PAR‐1 expression 26. However, the anticoagulant was ineffective as inhibitor of invasion in a Matrigel assay. This partial antineoplastic effect of LMWH may be explained by its weak ability to neutralize thrombin which is limited to plasma thrombin and not to tissue‐bound thrombin. Full neutralization can be accomplished by thrombin inhibitors 27, 28.

Lim et al. demonstrated that heparan sulfate and heparin may inhibit PAR‐1 activation by displacing binding to thrombin and thereby impairing carcinoma cell collagen invasion and degradation and attenuation of invasiveness of MDA‐MB231 cells 29.

In our model, thrombin activates PAR‐1 which enables signaling eventually promoting tumor progression. However, this model does not entirely define the role of PAR‐1 in cancer progression. In fact, there is evidence that the expression of the receptor by itself is sufficient to promote growth and invasion of tumor xenografts in nude mice and that knocking down PAR‐1 gene expression impaired the mobility of invasive breast cancer cells. Therefore, we cannot entirely predict than dabigatran will be effective in an in vivo setting of PAR‐1 expressing cancers as signaling could be thrombin‐independent 14. In these thrombin‐independent, PAR‐1‐driven tumor progression models, the addition of the protease or peptide agonists unexpectedly led to inhibition of invasion and migration of tumor cells. Other authors have reported an inhibitory effect of thrombin in the growth of several tumor cell lines 30. This is only partially in contrast with our results as thrombin concentration may affect tumor cell behavior differently as we found that increasing concentrations of thrombin negatively affected the chemotactic migration of tumor cells in vitro. Therefore, it is difficult to hypothesize the relevance of thrombin in the tumor microenvironment in term of tumor growth promotion.

Our study has obviously several limitations. A negative control with U87‐MG and MDAMB231 cells not expressing PAR‐1 would have further strengthened the relevance of the crosstalk. However, this may be not an optimal control as PAR‐1 silencing may affect basal cell growth. Also, we have no data on the effect of heparins or other anticoagulants in our system. With regard to heparins, one may predict that UF and LMWH can strongly bind thrombin 31 and it would have been interesting to explore whether this is sufficient to displace thrombin from PAR‐1 binding and therefore to affect PAR‐1 signaling.

We found dabigatran was effective at a concentration that is close to the one typically achieved in patients 32. However, extrapolation of a similar effect in vivo is unfeasible as thrombin concentration in the tumor microenvironment is difficult to predict. Our in vitro experimental setting therefore not necessarily reflects the complexity of a biological system. Moreover, the relevance of thrombin model in cancer biology is multifaceted and not univocally consistent. Our results may support the rationale to study the impact of dabigatran in the progression of cancer in patients with cancer and thrombosis. Alternatively, the well‐known data from Shulman and Miller, 33, 34 suggesting that thrombin may contribute to preserve tumor dormancy, may provide an appealing background for testing thrombin antagonists as an adjuvant treatment of patients with newly diagnosed cancer or for studying the development of cancer in patients with idiopathic DVT.

## Conflict of Interest

While there are no perceived conflicts of interest related to the work described in this manuscript, F.V. disclose that this study was partially supported by Boehringer Ingelheim Pharma GmbH & Co. KG.
